# Supplementary material for: Modelling cognitive outcomes in the UK Biobank: Education, noradrenaline and frontoparietal networks
Source: PLoS One. 2026 Jun 4;21(6):e0350452. doi: 10.1371/journal.pone.0350452 (PMC13235897; doi:10.1371/journal.pone.0350452)
Supplement: S1 Fig — A) Missing values pattern exploration using R package UpSetR showing the combinations of missingness across cases. B) Missing values pattern exploration using R package mice and finalfit. Missing values in red and complete in blue, with total numbers of participants found to the left and total missing features (red squares) to the right. Total missing values per feature found at the bottom. (PDF) [file pone.0350452.s001.docx]

**UK Biobank ID** **Fields used**

**Demographic information and cognitive data**

f.34.0.0:Clinical_Birth_Year

f.52.0.0:Clinical_Birth_Month

f.53.2.0:Clinical_Date_Assessment

f.31.0.0:Clinical_Sex

f.845.0.0:Education_0

f.845.1.0:Education_1

f.845.2.0:Education_2

f.20023.2.0:Cog_ReactionTime

f.4282.2.0:Cog_NumericMemory

f.20016.2.0:Cog_FluidIntelligence

f.6348.2.0:Cog_TrailA

f.6350.2.0:Cog_TrailB

f.6373.2.0:Cog_MatrixPatterns

f.21004.2.0:Cog_TowerRearr

f.23324.2.0:Cog_SymbolSubs

f.20197.2.0:Cog_PairedAssoc

f.399.2.2:Cog_PairMatch

**MRI data**

f.25661.2.0:WM_ICVF_ForcepsMinor

f.25662.2.0:WM_ICVF_IFOF_Left

f.25663.2.0:WM_ICVF_IFOF_Right

f.25671.2.0:WM_ICVF_SLF_Left

f.25672.2.0:WM_ICVF_SLF_Right

f.25688.2.0:WM_OD_ForcepsMinor

f.25689.2.0:WM_OD_IFOF_Left

f.25690.2.0:WM_OD_IFOF_Right

f.25698.2.0:WM_OD_SLF_Left

f.25699.2.0:WM_OD_SLF_Right

The 25 components group-ICA spatial maps and connection edges are available online (http://www.fmrib.ox.ac.uk/ukbiobank).

c7, c11, c15, c16, c20, c21, c29, c33, c34, c35, c106, c110, c111, c112,c114
