# Supplementary figures and images for: Modelling cognitive outcomes in the UK Biobank: Education, noradrenaline and frontoparietal networks
Source: PLoS One. 2026 Jun 4;21(6):e0350452. doi: 10.1371/journal.pone.0350452 (PMC13235897; doi:10.1371/journal.pone.0350452)

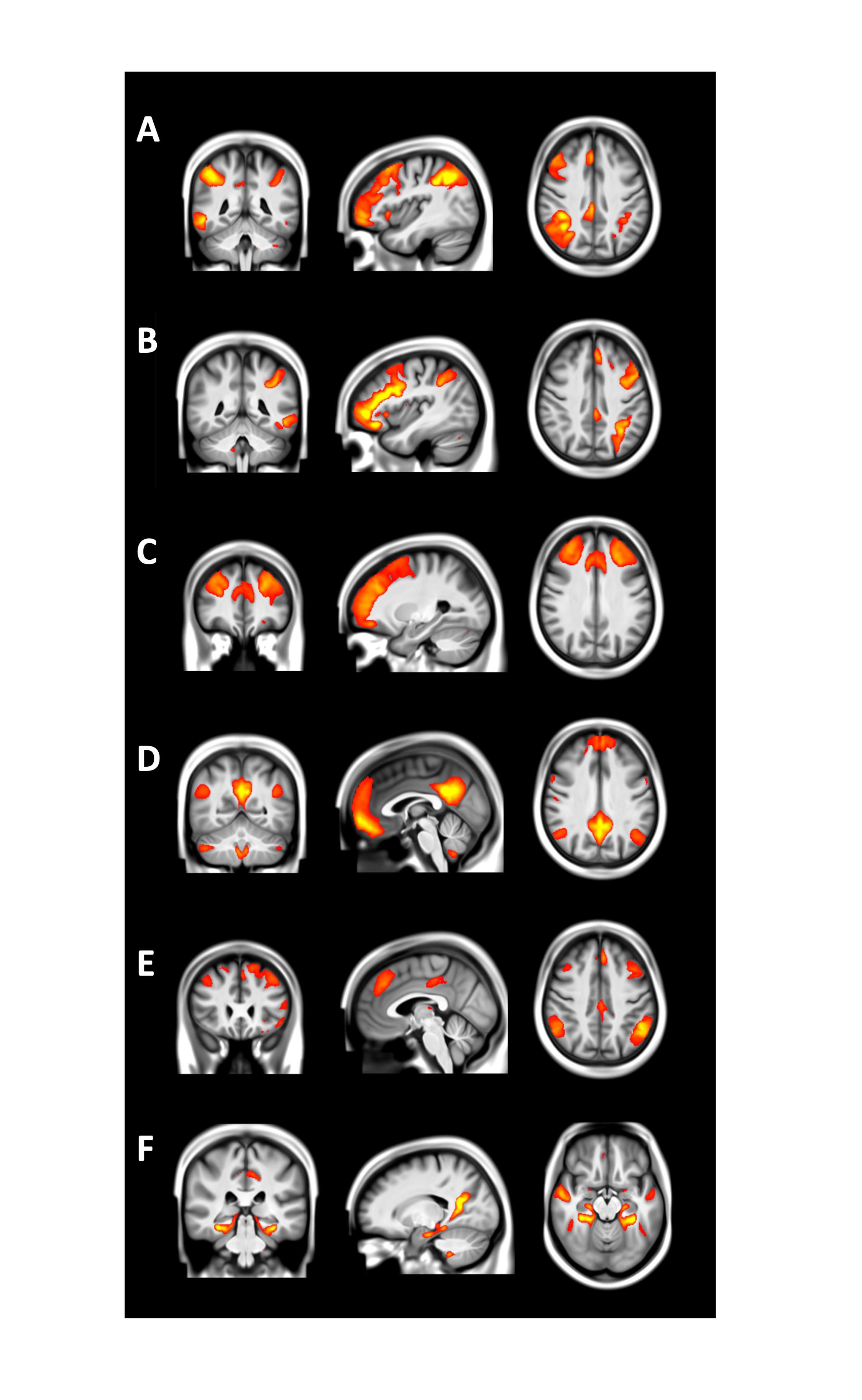

Supplement: S2 Fig — The six ICA components (nodes) representing restig state networks chosen for the purpose of the current study. A) right fronto-parietal network, B) left fronto-parietal network, C) executive control network and 3 subsytems of the default mode network: D) core (cDMN), E) dorsomedial prefrontal (dmDMN) and F) medial temporal (mtDMN). (TIFF) [file pone.0350452.s002.tiff]

A

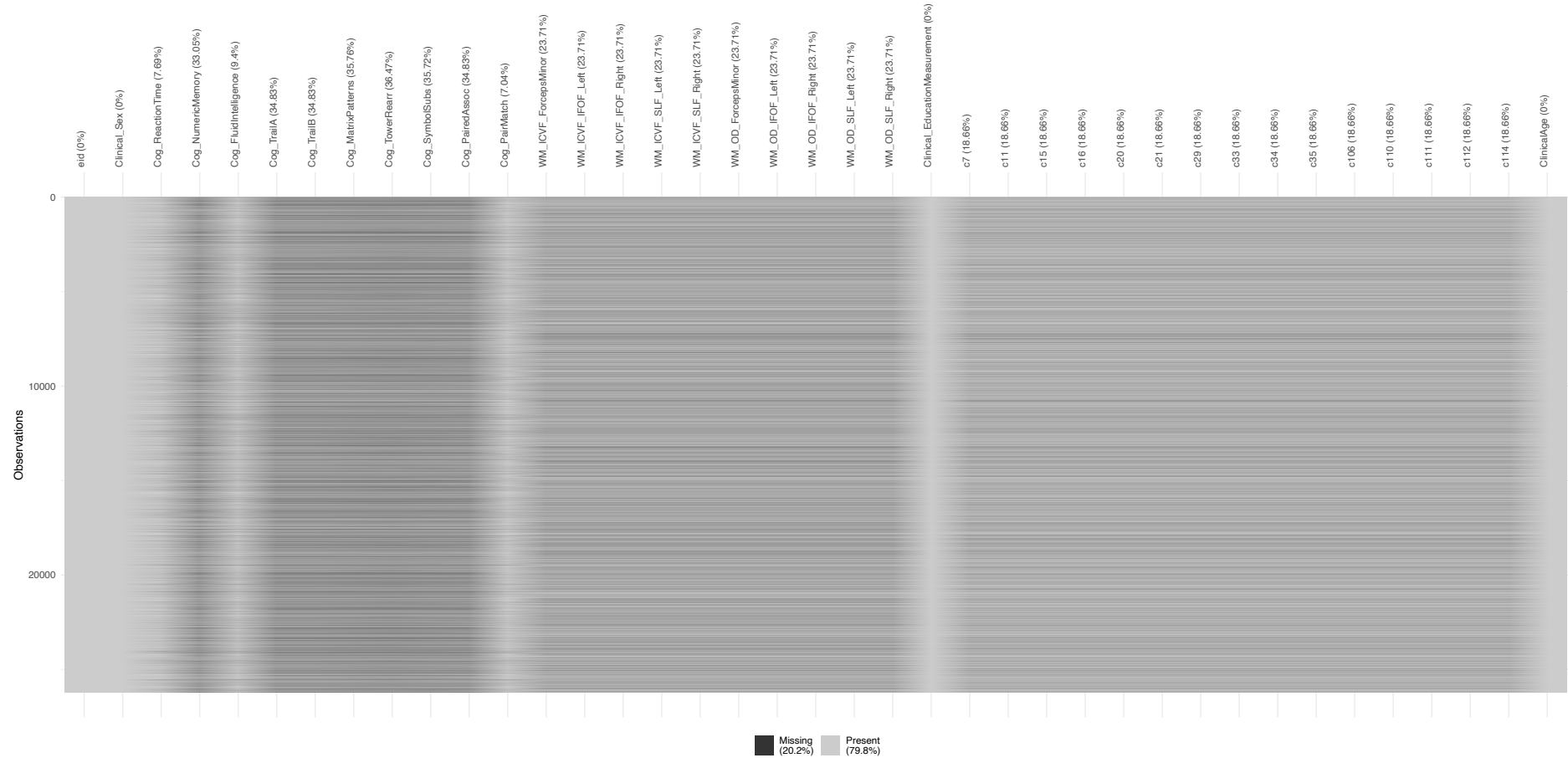

B

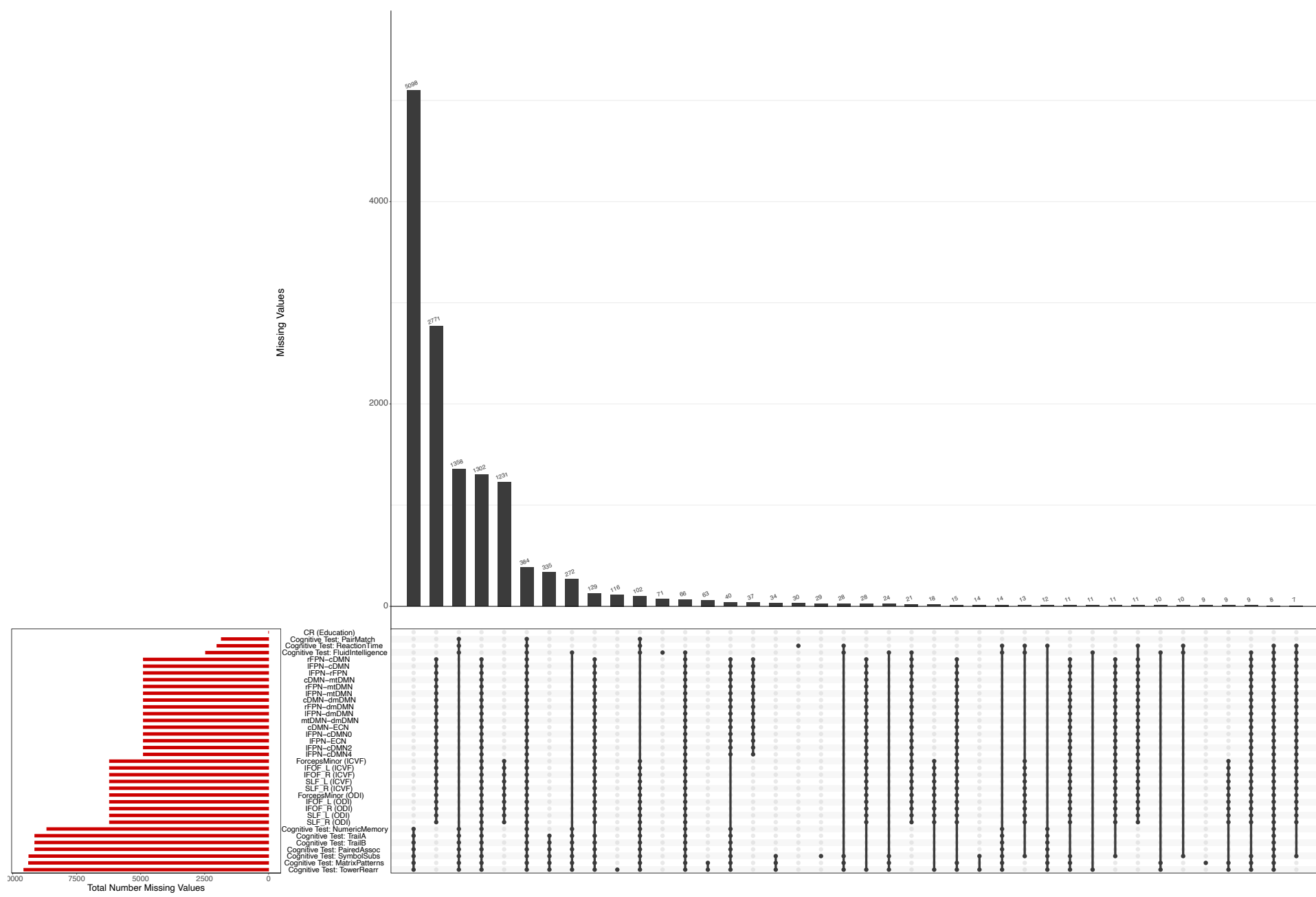

Supplement: S3 Fig — Missing values pattern exploration using R package UpSetR and naniar, showing the combinations of missingness across cases. Maximimum of missing cases per column is the cognitive test of tower rearrangement with (36.47%) missing data. (PDF) [file pone.0350452.s003.pdf]

Scree plot

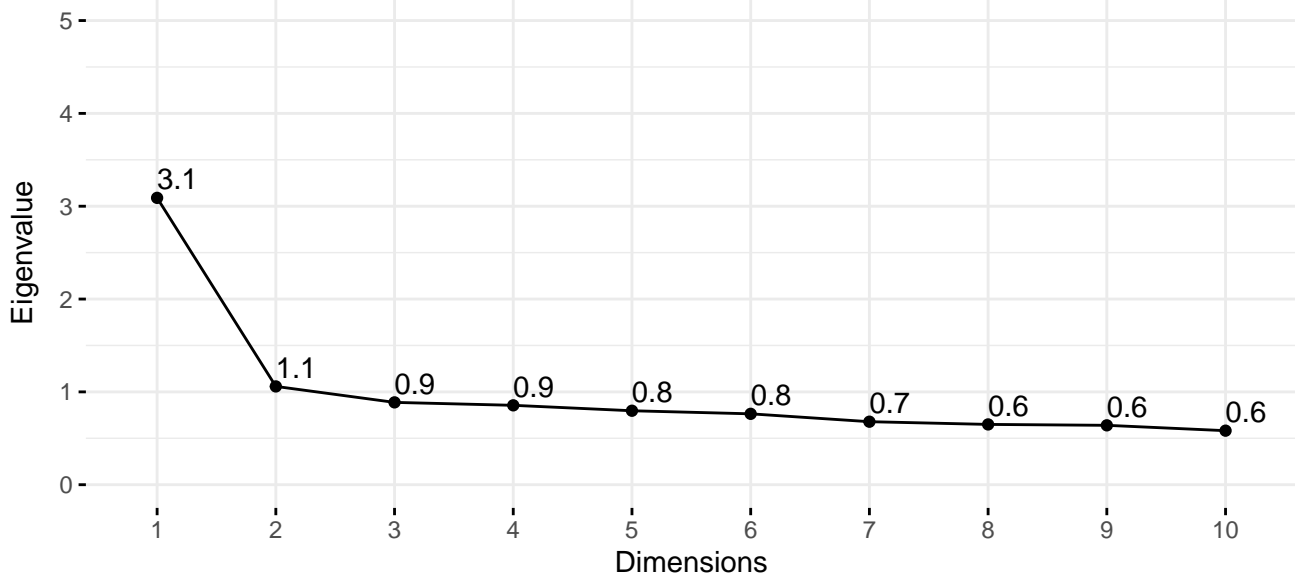

Scree plot

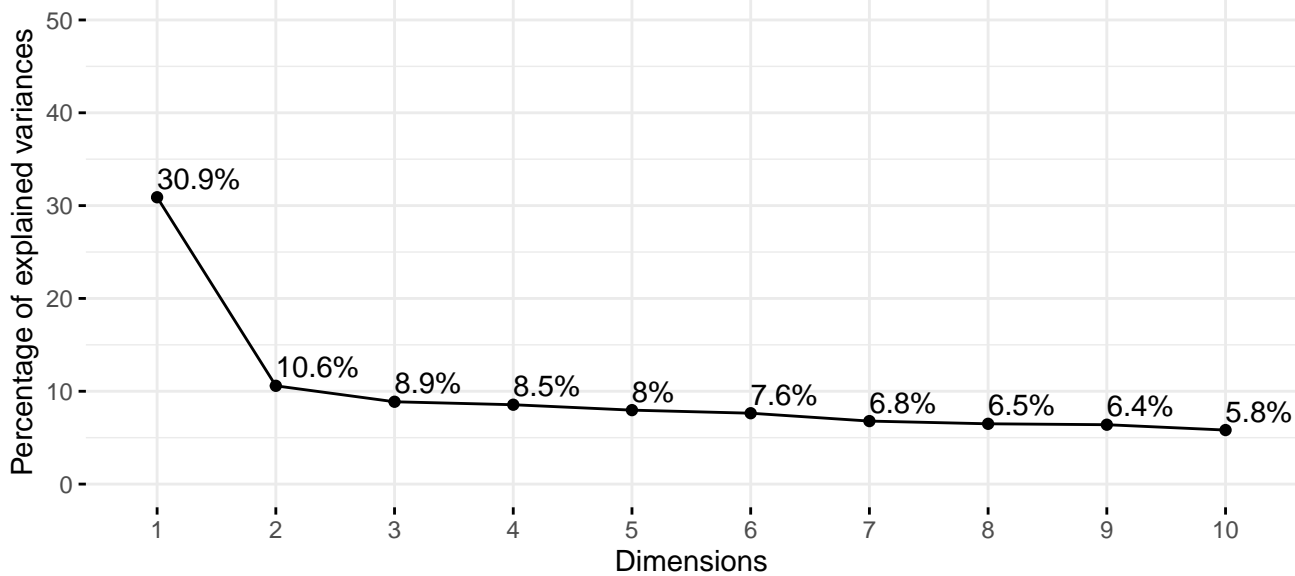

Supplement: S4 Fig — See Methods section for further information. (PDF) [file pone.0350452.s004.pdf]

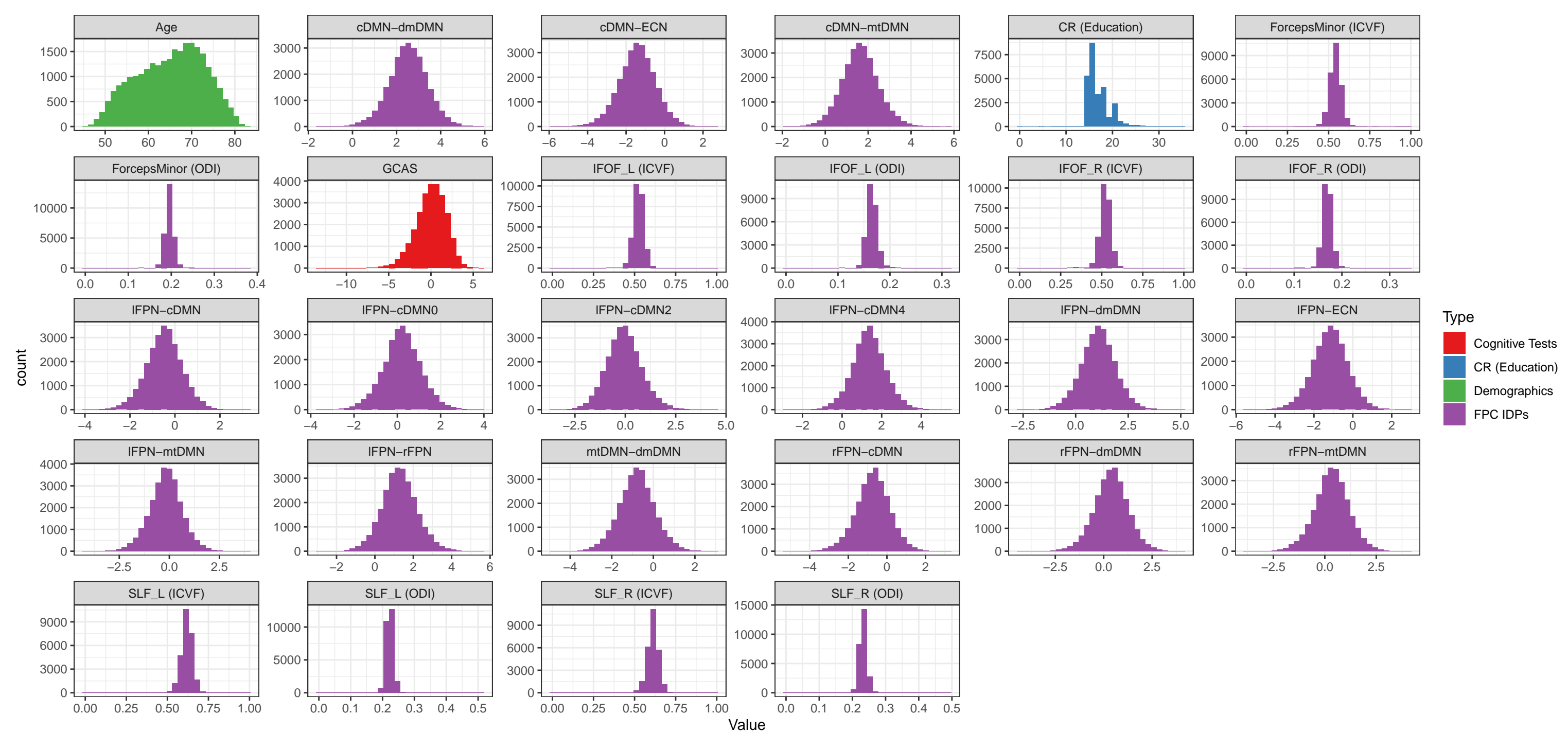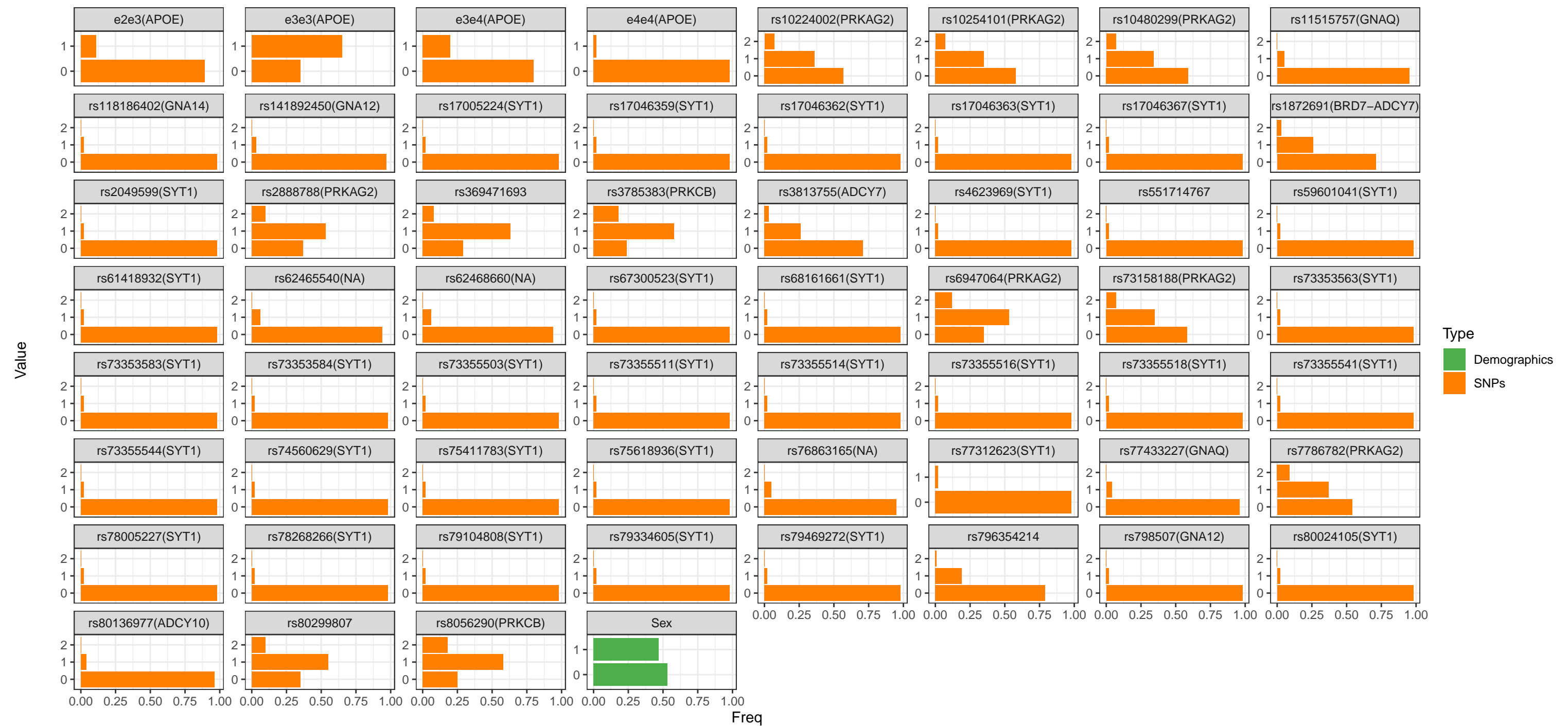

Supplement: S5 Fig — Continuous and categorical distribution of selected features with information on cognitive tests, demographics, education, genes and multimodal neuroimaging data. (PDF) [file pone.0350452.s005.pdf]

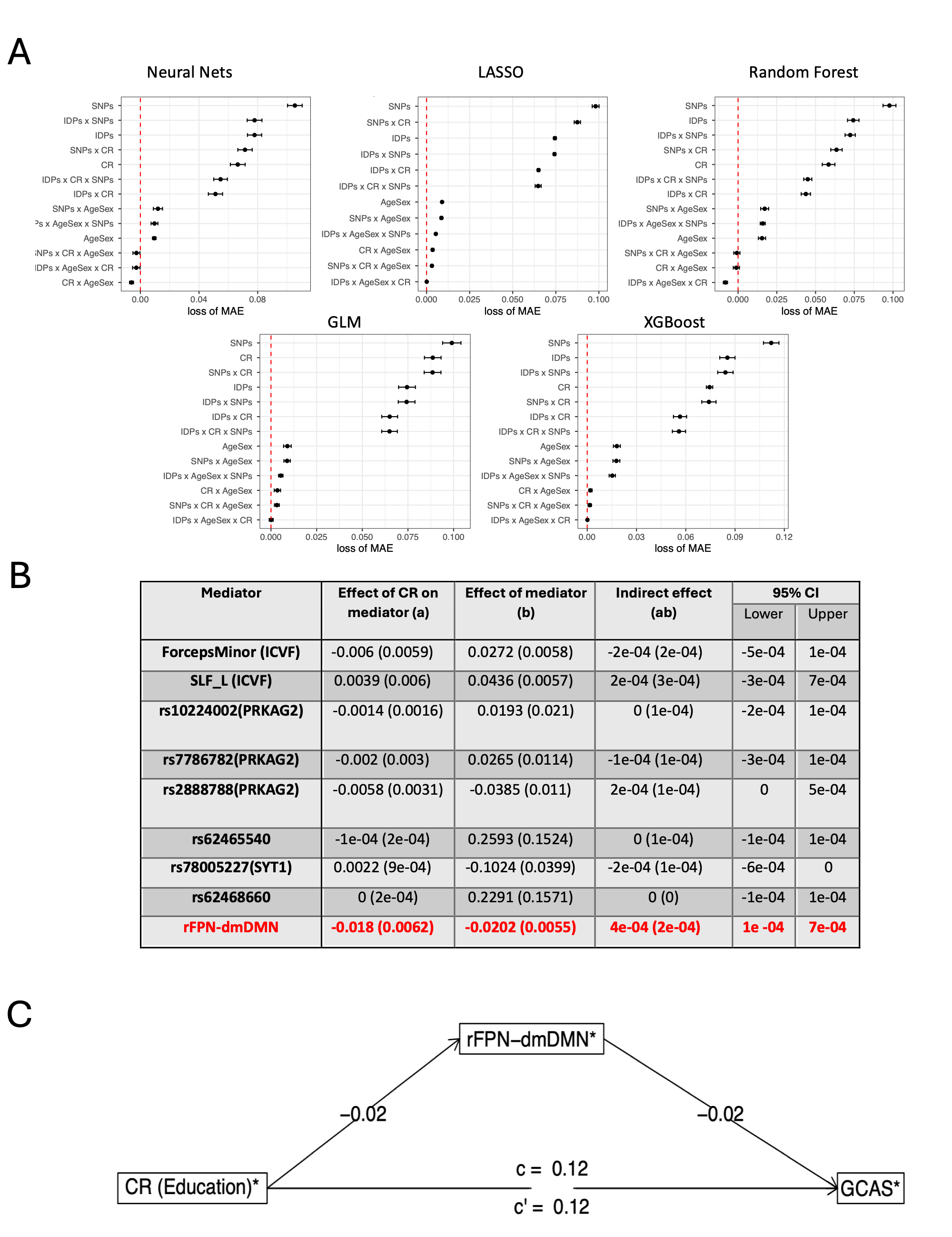

Supplement: S6 Fig — Imputed Dataset (K-nearest neighbours using 3 neighbours in tidymodels) given that only 20.2% of our dataset is missing but 54% (14,151/26,227) of participants are deleted from our analysis in complete case analysis, we performed a sensistivity analysis with imputed data to understand how and if results differed substantially with more data. A) Subsequently using imputation data, same analaysis was performed and the combination of IDPs, AgeSex and CR came as best performing too in most algorithms as is the case for the complete case analysis (as in Figure 2). Subsequently, feature importance was assessed in the same way as in complete case analysis for top performing features. Please note, 17 features differed (8 SNPs only selected in imputed data analysis and 9 IDPs and SNPs only found in complet-case analysis, with 14 chosen in common). B) Mediation results using for top 9 most frequent important variables. C) In mediation analysis, rFPN-dmDMN connectivity was found as a significant in both complete case analysis and imputed data analysis ascertaining the robustness of findings. (TIFF) [file pone.0350452.s006.tiff]
