# Supplementary material for: Modelling cognitive outcomes in the UK Biobank: Education, noradrenaline and frontoparietal networks
Source: PLoS One. 2026 Jun 4;21(6):e0350452. doi: 10.1371/journal.pone.0350452 (PMC13235897; doi:10.1371/journal.pone.0350452)
Supplement: S2 Table — PCA coordinates (loading* standard deviation) using factoextra. (DOCX) [file pone.0350452.s008.docx]

|  | PC 1 | PC 2 | PC 3 |
| --- | --- | --- | --- |
| ReactionTime | -0.384 | 0.496 | -0.473 |
| NumericMemory | 0.512 | 0.429 | 0.084 |
| FluidIntelligence | 0.636 | 0.412 | 0.018 |
| TrailA | -0.544 | 0.379 | -0.069 |
| TrailB | -0.587 | 0.17 | -0.076 |
| MatrixPatterns | 0.646 | 0.185 | 0.01 |
| TowerRearr | 0.626 | 0.002 | -0.077 |
| SymbolSubs | 0.652 | -0.231 | 0.056 |
| PairedAssoc | 0.495 | 0.33 | 0.006 |
| PairMatch | -0.395 | 0.3 | 0.798 |
